# Axillary web syndrome following secondary breast-conserving surgery: a case report

**DOI:** 10.1186/1477-7819-11-8

**Published:** 2013-01-17

**Authors:** Panmei Wei, Liling Zhu, Kai Chen, Weijuan Jia, Yue Hu, Fengxi Su

**Affiliations:** 1 Department of Breast Surgery, Sun Yat-sen Memorial Hospital, Sun Yat-sen University, Guangzhou, 510120, PR China

**Keywords:** Axillary web syndrome, Breast cancer, Sentinel lymph node biopsy, Thrombophlebitis

## Abstract

**Background:**

Axillary web syndrome is a cause of significant morbidity in the early postoperative period after axillary surgery.

**Case presentation:**

A patient developed axillary web syndrome after secondary breast surgery and recovered in 3 weeks through physical therapy and using Aescuven Forte.

**Discussion:**

The pathogenesis of axillary web syndrome is not clear. It is reported that axillary surgery is the main cause. The presented case indicates that tissue injury might be an important cause of axillary web syndrome. Though axillary web syndrome is self-limiting, special physical therapy and Aescuven Forte can shorten the natural duration.

**Conclusion:**

Secondary breast surgery could cause axillary web syndrome. Physical therapy and Aescuven Forte could shorten the duration of the self-limited morbidity.

## Background

Axillary web syndrome (AWS), which is characterized by visible or palpable cord-like subcutaneous tissue, primarily develops under the axilla but can extend into the medial arm and even down to the antecubital fossa and wrist. It is a cause of significant morbidity in the early postoperative period after axillary surgery. It limits the range of motion (ROM) of the shoulder, causing numbness, pain and tightness. The pathogenesis of AWS is not clear, and a few case reports and studies on this subject have demonstrated that AWS is a self-limiting benign occurrence; no treatments have been proven to be effective. To illustrate this poorly understood clinical syndrome, we present a case of AWS developing after secondary breast-conserving surgery.

## Case presentation

A 39-year-old otherwise healthy woman was diagnosed with breast cancer by mammography and underwent breast-conserving surgery and axillary lymph node biopsy for node-negative breast cancer in December 2011. The patient recovered without postoperative sequelae via conventional treatment, including antibiotics and exercise, during the postoperative period.

During the breast-conserving surgery, we performed cavity margin assessments to achieve margin clearance [1]. However, on the 17^th^ day after the first surgery, the patient had to undergo secondary breast surgery on only the breast, without lymph-node dissection, to achieve negative margins by excising seven margins. This was because the postoperative pathology report indicated a diagnosis of ductal carcinoma *in situ*; however, only two of the five margins excised in the first surgery were diagnosed with ductal carcinoma *in situ*, whereas two were diagnosed with severe atypical hyperplasia. Three days after the secondary breast surgery, the patient complained of severe pain in the ipsilateral axilla and interior aspect of the arm after shoulder abduction and a subcutaneous cord-like structure in the axilla.

**Figure 1:**
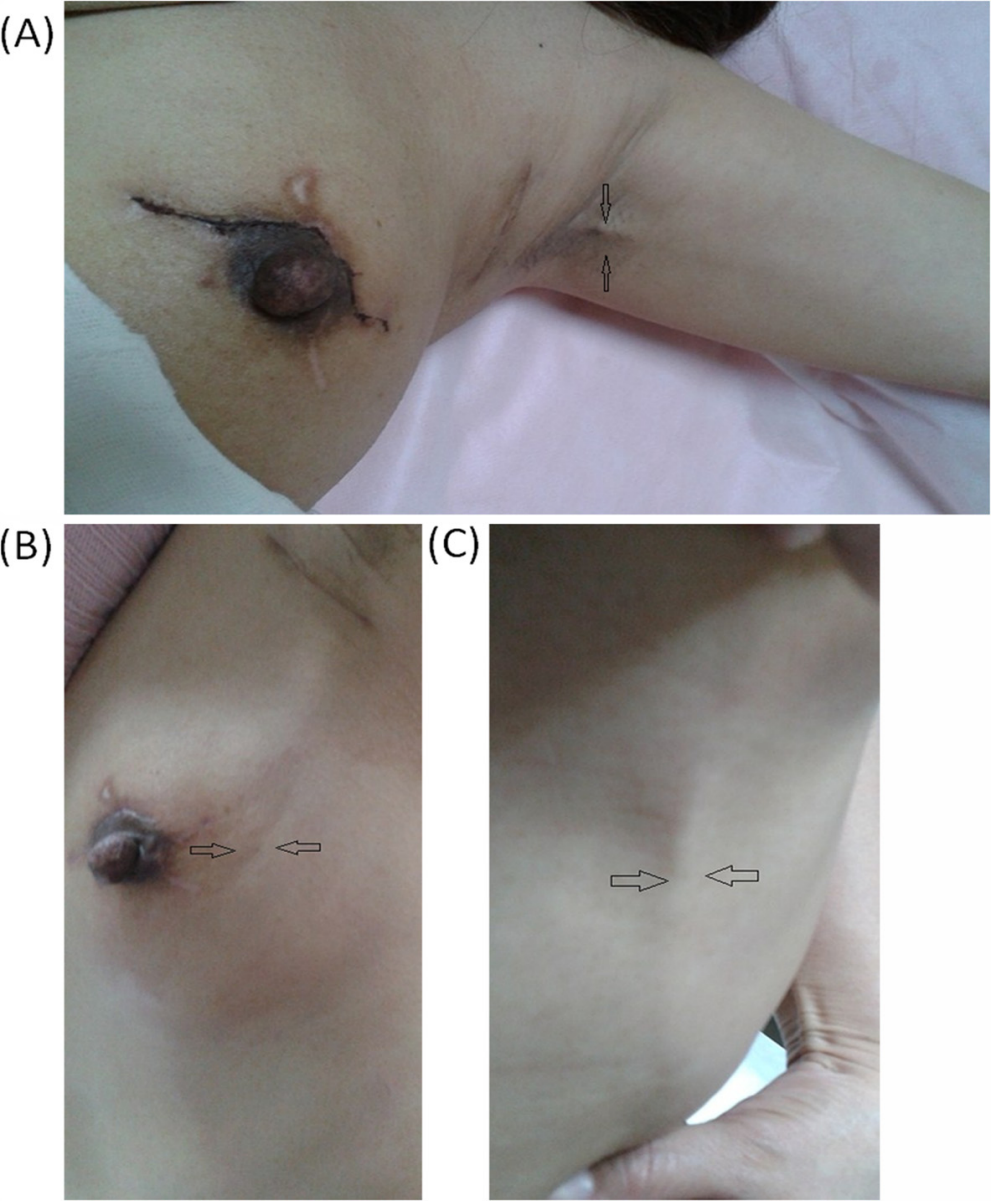
**Physical examination of patient with axillary web syndrome. (A)** Patient demonstrates axillary web syndrome in the left arm. Taut cords extending from the axilla to the medial arm are observed. The patient is unable to fully abduct her left shoulder. **(B)** Taut cords radiate to the upper outer quadrant of the breast. **(C)** Taut cords radiate to the thoracoabdominal wall.

**Figure 2:**
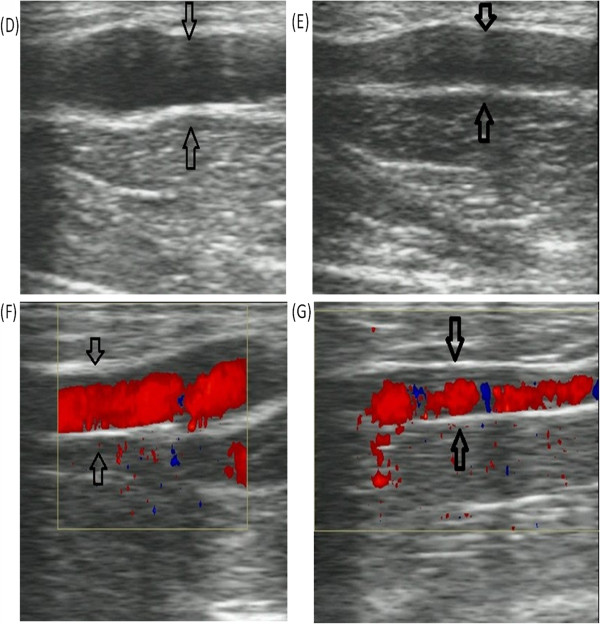
**B-type ultrasonography of the cords extending from the axilla to the medial arm. (A)** The cords under the axilla present Eco of a tube structure. **(B)** The cords in the medial arm present the echo of a tube structure. **(C, D)** Blood is observed in the tube structures of (**A**) and (**B**), respectively.

A physical examination found visible and palpable cords (string-like) extending from the axilla to the medial arm on ipsilateral shoulder abduction (Figure 1A). No erythema, warmth or any other inflammatory signs were observed in the patient. The patient complained of severe pain when abducting her left shoulder and her shoulder ROM was reduced from 175° (preoperative) to 90°, as measured by a goniometer. She gave her pain a score of 7 using the Visual Analogue Scale, which is quick, reliable and valid for measuring pain and pain relief [2,3]. During follow-up, the patient complained that the cords had extended to the elbow and the ipsilateral upper outer quadrant of the breast, and her thoracoabdominal wall was also involved (Figure 1B,C). Numbness and tightness were present during the entire follow-up. B-type ultrasonography indicated that the subcutaneous cords that radiated from the axilla to the medial arm presented an obvious blood flow signal on color Doppler flow imaging, which proved that the cord-like structure was a blood vessel (Figure 2A-D), whereas the cords that radiated to the breast and thoracoabdominal wall presented the same echo of subcutaneous tissue as the contralateral thoracoabdominal wall without cords.

Because the patient complained of severe pain and limited shoulder ROM, we treated her with 300 mg of Aescuven Forte twice a day for one week and advised her to perform shoulder exercises, including abducting the shoulder and massaging the cord-like structure for 30 minutes every day in the morning and at bedtime as soon as the AWS occurred. After two weeks of follow-up, the patient presented with pain-free shoulder abduction, a ROM of 150°, and cords that had become smaller and not as significant. Three weeks later, the patient returned for a physical examination and could abduct her shoulder, with a ROM of 170°, without any pain. However, numbness and tightness were still present but somewhat diminished. The cords were invisible and non-palpable.

## Discussion

AWS can develop during many conditions and is considered to be a type of Mondor’s disease, characterized by a thick-wall blood vessel or lymphatic vessel [4,5]. It has been attributed to prior trauma or breast surgery, such as breast reduction, mammoplasty or lumpectomy [6]. Moskovitz*et al.*[7] coined the term AWS and proposed that the pathogenesis is lymphovenous damage, hypercoagulation, superficial venous and lymphatic stasis, and disorders and injuries of tissues as a result of the disruption of superficial lymphatic and blood vessels during axillary surgery. Our patient developed AWS after secondary breast surgery without lymph-node dissection to achieve negative margins, which suggests that it was the secondary breast surgery that promoted development of AWS rather than the original axillar lymph-node biopsy. This differed from previous studies, in which stress from the axillar lymph-node dissection or axillar lymph-node biopsy caused AWS [8,9]. We propose that the tissue injury caused by the secondary surgery led to the release of inflammatory factors, which caused phlebitis via intravasation in the axilla, medial arm, breast and thoracoabdominal wall through multiple mechanisms. The hypothesis is consistent with the B-type ultrasonography findings, but was not definitive due to the lack of a histopathology report. Most of the investigators were inclined to define the cord-like structure as a lymph vessel [7,8,10,11].

AWS is a self-limiting cause of morbidity in the early postoperative period following axillary surgery [7,8].There are no standard therapeutic methods reported for AWS. Previous studies have indicated that physical therapy can shorten the natural course of AWS to 6 to 8 weeks [7,8,12]. In this case, we recommended that the patient perform physical therapy to alleviate the symptoms and treated her with Aescuven Forte, which is used to treat phlebitis in the clinic. After the interventions, the duration of AWS in this case was shortened to 3 weeks, which suggests that Aescuven Forte might be effective in improving AWS in combination with physical therapy. However, we cannot determine exactly which therapeutic method had the better effect on the alleviation of AWS. Therefore, more clinical data should be collected to study the therapy of AWS.

In summary, AWS is a self-limited disease that presents typical symptoms of pain, tightness, restriction of shoulder ROM and a subcutaneous cord-like structure. It is thought to be caused primarily by axillary lymph node dissection or sentinel lymph node biopsy. In addition to the physical symptoms, AWS also leads to patient anxiety and fear due to a poor understanding of the complication, which affects the patient’s quality of life. However, many surgeons learn little about and usually overlook this complication and seldom put the patient on appropriate treatment. Consequently, we need to learn more about the pathogenesis, histopathology and effective therapy for AWS. To further investigate AWS, more prospective clinical studies are warranted.

## Conclusion

Secondary breast surgery could cause AWS. Physical therapy and Aescuven Forte could shorten the duration of the self-limited morbidity.

## Consent

Written informed consent was obtained from the patient for publication of this Case report and any accompanying images. A copy of the written consent is available for review by the Editor-in-Chief of this journal.

## Abbreviations

AWS: Axillary web syndrome; ROM: Range of motion.

## Competing interests

The authors declare that they have no competing interests.

## Authors’ contributions

PW and YH performed the examination; PW, LZ and KC wrote the paper; FS and WJ performed the surgery. All the authors reviewed and approved the final manuscript.
